# Optimized amyloid‐β PET centiloid and cognitive cutoffs predict progression from mild cognitive impairment to Alzheimer's dementia

**DOI:** 10.1002/alz.71892

**Published:** 2026-11-05

**Authors:** Rosita Shishegar, Vincent Doré, Pierrick Bourgeat, James D. Doecke, Rodrigo Cánovas, Simon M. Laws, Tenielle Porter, Michael Weiner, Colin L. Masters, Hamid R. Sohrabi, Jurgen Fripp, Paul Maruff, Victor L. Villemagne, Christopher C. Rowe, for the Alzheimer's Disease Neuroimaging Initiativethe Australian Imaging BiomarkersLifestyle flagship study of ageing

**Affiliations:** ^1^ The Australian e‐Health Research Centre CSIRO Melbourne Victoria Australia; ^2^ School of Computing Technologies RMIT University Melbourne Victoria Australia; ^3^ The Australian e‐Health Research Centre CSIRO Brisbane Queensland Australia; ^4^ Centre for Precision Health Edith Cowan University Joondalup Western Australia Australia; ^5^ Collaborative Genomics and Translation Group School of Medical and Health Sciences Edith Cowan University Joondalup Western Australia Australia; ^6^ Center for Imaging of Neurodegenerative Diseases University of California‐San Francisco San Francisco California USA; ^7^ Alzheimer's Research Australia University of Western Australia Nedlands Western Australia Australia; ^8^ Centre for Healthy Ageing Health Futures Institute Murdoch University Murdoch Western Australia Australia; ^9^ School of Psychology Murdoch University Murdoch Western Australia Australia; ^10^ Cogstate Ltd Melbourne Victoria Australia; ^11^ Department of Psychiatry University of Pittsburgh Pittsburgh Pennsylvania USA; ^12^ Florey Institute of Neuroscience and Mental Health The University of Melbourne Parkville Victoria Australia; ^13^ Department of Molecular Imaging & Therapy Austin Health Heidelberg Victoria Australia

**Keywords:** amyloid‐β PET, cognitive decline, disease progression risk, Mini‐Mental State Examination score, mild cognitive impairment, prognostic

## Abstract

**INTRODUCTION:**

This study aimed to develop optimized prognostic cutoffs for amyloid‐β–positron emission tomography (Aβ‐PET) and Mini‐Mental State Examination (MMSE) that predict 3‐year progression from Clinical Dementia Rating Global (CDR‐Global) score 0.5 to 1.0 in mild cognitive impairment (MCI) participants and validate against cognitive decline/functional trajectories.

**METHODS:**

We analyzed 454 CDR‐global = 0.5 Aβ‐positive (> 25 Centiloid [CL]) MCI participants from Australian Imaging Biomarkers and Lifestyle (AIBL) (*N =* 99) and Alzheimer's Disease Neuroimaging Initiative (ADNI) (*N =* 355), followed up to seven years. Youden index derived optimal Aβ‐PET and MMSE thresholds for 3‐year progression. Participants were stratified into four groups by combined Aβ‐PET/MMSE status. Adjusted Cox models (age, sex, education, apolipoprotein E [*APOE]* ε4) assessed progression to CDR‐Global score of 1.0 Alzheimer's disease (AD) dementia (confirmed with Aβ‐PET); adjusted linear mixed models assessed CDR Sum of Boxes (CDR‐SoB) and Preclinical Alzheimer's Cognitive Composite (PACC) trajectories.

**RESULTS:**

Optimal cutoffs were Aβ‐PET > 71 CL (hazard ratio [HR] = 2.67; 3‐year risk = 36.7%) and MMSE score of < 27 (HR = 2.27; 36.8%). The combined high Aβ/low MMSE group showed a 6.39‐fold risk and 47.8% 3‐year progression, with the steepest decline.

**DISCUSSION:**

Prognostically optimized Aβ‐PET and MMSE cutoffs improve 3‐year risk stratification beyond common diagnostic thresholds, supporting dual‐cutoff prognostic enrichment.

## Highlights

- Prognostic cutoffs of amyloid‐β–positron emission tomography (Aβ‐PET) > 71 Centiloid (CL) and Mini‐Mental State Examination (MMSE) score < 27 optimized for clinical progression.
- Combined cutoffs showed 6.39‐fold higher risk of progression to Clinical Dementia Rating Global (CDR‐Global) score of 1.0 Alzheimer's disease (AD) dementia.
- High‐risk group showed a 47.8% 3‐year progression rate (CDR‐Global score of 0.5 to 1.0).
- High Aβ‐PET > 71 CL drove rapid Preclinical Alzheimer's Cognitive Composite (PACC) decline independent of baseline MMSE score.
- Combined Aβ‐PET > 71 CL and MMSE score of < 27 predicted steepest decline for CDR Sum of Boxes (CDR‐SoB).

## 1 BACKGROUND

Recent therapeutic strategies for Alzheimer's disease (AD) ^1^, ^2^have aimed to reduce disease progression in the early symptomatic stages, including in mild dementia and mild cognitive impairment (MCI). Clinically, MCI differs from mild dementia in that, although both stages involve substantial cognitive and behavioral symptoms, these are less severe in MCI and impairments in activities of daily living occur only in the most demanding contexts. While early symptomatic AD can be identified reliably based on AD biomarker levels and standardized ratings of clinical or mental status, these same criteria have shown limited accuracy in predicting rates of disease progression in the individuals classified, with considerable heterogeneity characteristic of the early symptomatic AD samples.^2^, ^3^ This heterogeneity likely arises because the criteria used to classify MCI among cognitively unimpaired (CU) older adults, or to confirm amyloid‐β–positron emission tomography (Aβ‐PET) positivity, are binary and optimized for case dentification rather than prognosis.^4^, ^5^, ^6^ Consequently, developing criteria based on these AD characteristics, but optimized for predicting clinical progression, would be useful for enriching clinical trial samples, assisting clinicians in tailoring care plans (e.g., monitoring frequency), and informing evaluation of anti‐AD therapies.^7^, ^8^, ^9^

Previous studies have examined whether criteria for Aβ‐PET levels or mental state examination scores can be optimized to estimate risk of disease progression. However, their prognostic utility has been limited by studies seeking solutions for the complete AD clinical spectrum, rather than for prodromal AD specifically.^10^, ^11^ In addition, many studies have used relatively small samples (e.g., < 200 participants)^11^, ^12^ or have studied progression across brief follow‐up periods (e.g., 18–36 months).^12^, ^13^ Another limitation is that most studies have defined disease progression using the magnitude of change in cognition composite measures as their outcome and where these have been expressed mathematically on a continuous scale (i.e., on the Preclinical Alzheimer's Cognitive Composite [PACC]). While modeled rates of cognitive decline are instructive for AD clinical‐pathological models, they are less practical for clinical practice and lack validated thresholds for clinically meaningful change.^14^

**RESEARCH IN CONTEXT**

**Systematic review**: Existing amyloid‐β–positron emission tomography (Aβ‐PET) and Mini‐Mental State Examination (MMSE) cutoffs were developed to classify Alzheimer's disease (AD) pathology (e.g., Aβ‐PET positivity at 20–50 Centiloid [CL]), not to predict disease progression. Our reviewed literature showed that these classification‐derived diagnostic thresholds contribute to prognostic heterogeneity when applied to individuals with mild cognitive impairment (MCI).
**Interpretation**: We derived prognostically optimized cutoffs (Aβ‐PET > 71 CL; MMSE score of < 27) that predict 3‐year clinical disease progression (Clinical Dementia Rating Global [CDR‐Global] 0.5 to 1.0). Their combination enhanced risk prediction (6.39‐fold increased risk) beyond individual use. Validation against CDR Sum of Boxes (CDR‐SoB) and Preclinical Alzheimer's Cognitive Composite (PACC) trajectories confirmed that, above 71 CL, Aβ‐PET becomes the primary predictor of cognitive decline, independent of baseline cognitive performance.
3. **Future directions**: Future research should validate these cutoffs in diverse cohorts. Key questions include: (1) investigating interactions between these cutoffs and tau pathology spread; (2) whether prognostically refined criteria reduce heterogeneity in clinical trial design; (3) integration with emerging plasma biomarkers for accessible screening.

One approach to improve the prognostic utility of an Aβ‐PET biomarker and mental status information, individually and combined, is to examine outcomes in a large prospective study of adults with prodromal AD that has been classified at a baseline using both biomarkers and clinical examination and for whom disease progression has been monitored over a time period relevant to early‐stage disease management. To enhance clinical interpretability and generalizability of the criteria for risk of disease progression, progression can be defined as the worsening of clinical disease classification (e.g., progression from MCI to AD dementia). This approach also provides clinically meaningful anchor points for interpreting longitudinal changes in biomarkers and cognition measured on continuous scales.

The overarching goal of this study was to utilize prospective data on Aβ‐PET levels and clinical symptoms in adults with prodromal AD, defined as a clinical classification of MCI and a Aβ‐PET classification of Aβ+, from the Australian Imaging, Biomarkers, and Lifestyle (AIBL) and the Alzheimer's Disease Neuroimaging Initiative (ADNI) studies, to determine how baseline Aβ‐PET levels and mental status examination scores predict changes in these AD markers. Clinical disease progression was defined as occurring when global score on the Clinical Dementia Rating Global (CDR‐Global) increased from 0.5 to 1.0. This definition is used commonly in AD clinical trials for staging the transition from prodromal disease to dementia.^15^, ^16^ The first aim was to identify optimal levels of Aβ‐PET for predicting 3‐year clinical disease progression. The second aim was to evaluate whether cutoff for cognitive impairment defined from examination of mental status added prognostic value of that from the Aβ‐PET. The third aim was to determine the precision of the developed criteria for predicting clinical disease progression, and for predicting decline in cognition and function, defined using continuous outcomes.

## 2 METHODS

### 2.1 Participants

Data for the current study were derived from databases of participants enrolled in the AIBL^17^ and the ADNI^18^, ^19^ studies (for current information, see www.adni‐info.org). In both studies, the clinical classification of participants at each study visit was conducted by an independent review committee who had access to the study data. At entry to each study participants or their authorized carers had provided written informed consent. Data for this work were collected before the approval of anti‐amyloid treatment, and the participants were not exposed to the treatment. Further details about the study design can be found in previous publications.^17^, ^18^, ^19^

For the present study, participant data was selected from the AIBL and ADNI cohorts based on the first timepoint at which they met the following inclusion criteria: (1) age over 50 years; (2) presence of a subjective memory complaint accompanied by a CDR‐Global score of 0.5; (3) absence of severe dementia, defined as a Mini‐Mental State Examination (MMSE) score of 20 or higher; (4) availability of at least two neuropsychological assessments; and (5) evidence of AD biology, as indicated by Aβ‐PET positivity.

In the current study, the necessary inclusion criterion for Aβ‐PET positivity was Centiloid (CL) > 25. To determine this, PET datasets were processed using specialized pre‐processing systems designed for each cohort to quantify Aβ accumulation on the CL scale.^20^ All Aβ‐PET images were spatially normalized using CapAIBL software.^21^ The images were normalized in standardized uptake value ratio images using the whole cerebellum as reference region, before being quantified into CL using the Non‐negative Matrix Factorization approach^22^ which was previously proposed to harmonize CL quantification across the ADOPIC studies. Subjects who did meet all criteria, including the minimum of two follow‐ups over a maximum period of 7 years, were excluded. A total of 454 individuals met these criteria, including 355 from the ADNI cohort and 99 from the AIBL cohort.

### 2.2 Clinical and demographic information

Demographic information included for age, sex, years of education, and apolipoprotein E genotype (*APOE)* ε4 carrier status,^23^, ^24^, ^25^ Follow‐up duration and visits and cohort is presented in Table 1. *APOE* ε4 carriers were defined as participants with at least one ε4 allele (ε2/ε4, ε3/ε4, and ε4/ε4), while non‐carriers were participants without ε4 (e.g., ε2/ε2, ε2/ε3, ε3/ε3). Clinical information was based on cognitive data, including CDR‐Global score. The consensus clinical classification of participants based on the approach used in each study was applied here to define the clinical disease classification of each participant at each visit and, therefore, to provide a basis for the definition of clinical disease progression.

**TABLE 1:** Participant baseline characteristics by progression status

Parameter	Progressors	Non‐progressors	*p‐value*
n	93	361	
Cohort, n (%) ADNI	77 (82.8%)	278 (77.0%)	0.287
Age, years	74.6 ± 6.4	74.8 ± 7.3	0.76
Sex, n (%) female	41 (44.1%)	156 (43.2%)	0.973
*APOE* ε4 – positivity, %	70 (75.3%)	211 (58.4%)	0.00425
Education, years	15.4 ± 3.3	15.1 ± 3.2	0.342
Follow‐up duration, years	1.7 ± 0.8	3.5 ± 2.0	<0.001
Follow‐up visits, number	3.1 ± 1.0	4.5 ± 1.9	<0.001
Aβ‐PET, baseline value	91.7 ± 28.5	73.1 ± 32.1	<0.001
MMSE, baseline score	26.6 ± 2.0	27.5 ± 2.0	<0.001

*Note*: Continuous variables are indicated as follows: mean ± SD; ANOVAs were used for continuous variables and chi‐squared tests were used for categorical variables.

Abbreviations: Aβ‐PET, amyloid‐β–positron emission tomography; ADNI, Alzheimer's Disease Neuroimaging Initiative; ANOVA, analysis of variance; APOE, apolipoprotein E; MMSE, Mini‐Mental State Examination; SD, standard deviation.

### 2.3 Neuropsychological evaluation

In both the AIBL and ADNI studies, different batteries of neuropsychological tests have been used to measure cognitive domains known to be deteriorate most in early symptomatic AD. These have been described previously.^17^, ^18^, ^19^ The data from these different neuropsychological test batteries have been harmonized recently using an imputation‐based harmonization method to merge data for tests of the same cognitive function. The harmonization method uses random forests iteratively to impute unmeasured test scores in one dataset using the patterns of the test scores in the other dataset (described in references^26^, ^27^). For this study, a measure of cognition was generated by combining of scores from the harmonized data for verbal list learning, paragraph, processing speed and the MMSE scores, standardized using the mean and SD of a group Aβ‐PET negative adults who were CU from the same harmonized cohort, to provide a PACC composite score.^26^, ^27^ Clinical disease severity at each assessment was defined with the CDR Sum of Boxes (CDR‐SoB).^28^

### 2.4 Statistical analyses of demographic and clinical characteristics

Statistical analyses were performed using RStudio, version 2023.06.1, R version 4.3.1. Based on a prospective review of clinical disease status over time, participants with MCI (CDR‐Global score = 0.5 at baseline) were grouped by clinical disease progression. Those who showed disease progression within 3 years were classified as “progressors”. Those whose CDR‐Global scores remained at 0.5 over the study period were classified as “non‐progressors”. Differences between these two groups were assessed with independent t‐test for continuous data (e.g., age, years of education, and follow‐up duration) and the χ2 tests for categorical data (e.g., gender and *APOE* ε4 status).

### 2.5 Development of a criterion value for prediction of clinical disease progression

Cox proportional hazards models were utilized to analyze the progression from MCI due to AD (defined by a CDR‐Global score of 0.5; CL > 25) to mild AD (defined by a CDR‐Global score of 1). Survival time was defined as the duration from baseline until progression to mild AD, withdrawal from the study, or the last follow‐up visit, whichever occurred first. Cox models used the following reference categories: female, *APOE* ε4 non‐carrier status, mean age across all subjects, mean years of education across all subjects, and the low‐risk group. All Cox models were adjusted for age, sex, years of education, and *APOE* ε4 carrier status, as these are well‐established risk factors for AD progression. The Progression Risk (PR) = 1‐survival probability was calculated at 3 years using Cox proportional hazards models for each group.

To identify the optimal values and to distinguish groups with high‐risk and low‐risk for clinical disease progression, participants were initially classified based on their Aβ‐PET load. A range of cutoffs (from 30 to 95 CL) were tested, and the optimal cutoff was derived using the Youden index, ensuring the highest discrimination between the high‐risk and the low‐risk groups. Subsequently, the same statistical procedure was repeated using MMSE scores, with cutoffs tested across the scores between 20 to 30. We primarily report the 3‐year survival probability for several convergent reasons. First, a 3‐year horizon is clinically relevant to the duration of contemporary AD trials. Phase 3 trials in early symptomatic AD, including TRAILBLAZER‐ALZ 2 and CLARITY AD, evaluated their primary outcomes over approximately 18 months, whereas longer prevention trials in preclinical AD, such as AHEAD 3‐45, extend follow‐up to approximately 4 years.^1^, ^29^, ^30^ The 3‐year horizon, therefore, falls within the timeframe over which enrichment strategies must demonstrate prognostic utility to inform participant selection and trial design. Second, within our dataset, the numbers of participants at risk and observed progression events remained sufficient at 3 years to support reasonably precise Cox model estimates. At longer follow‐up durations, particularly beyond 5 years, the at‐risk population decreased substantially, resulting in wider confidence intervals (CIs) and reduced precision. Overall, the 3‐year time point provided an appropriate balance between clinical relevance, statistical precision, and the availability of follow‐up data.

Finally, participants were stratified into four risk groups based on the combination of the optimal cutoffs identified for Aβ‐PET load and MMSE scores: (1) low‐risk (reference group), (2) intermediate‐risk with impaired‐cognition, (3) Intermediate‐risk with high Aβ, and (4) highest‐risk group. The survival probabilities of individuals in the highest‐risk groups, as determined by these criteria (Aβ load, MMSE score, or their combination), were compared over a 7‐year period.

A nonparametric bootstrap resampling approach with 1000 iterations was applied to determine optimal biomarker cutoffs and assess their stability. For each iteration, we generated a new dataset by random sampling with replacement from the original data, maintaining the original sample size. The same statistical analysis was performed on each bootstrap sample, and optimal cutoffs were identified. A 95% CI was calculated using the 2.5th and 97.5th percentiles of the bootstrap distribution. To ensure validity, we enforced a minimum group size of 20 subjects per risk category. Bootstrap samples were validated using the Kolmogorov–Smirnov test to confirm that the distributions of survival time in high‐risk and low‐risk groups were statistically similar to those in the original dataset.

To assess the potential impact of cohort imbalance, we conducted cohort‐stratified sensitivity analyses and formal cohort‐by‐cutoff interaction tests; details are provided in the Supplementary Materials.

### 2.6 Validation analysis: Independent prognostic value of risk groups

To investigate whether the proposed Aβ‐PET/MMSE risk groups provide significant prognostic value beyond established clinical covariates (age, sex, *APOE* ε4 status, and years of education), we performed a Likelihood Ratio Test (LRT) comparing a baseline model containing only these clinical factors to an expanded model that incorporated the Aβ‐PET/MMSE risk groups. Furthermore, we assessed hazard ratio (HR) stability by comparing unadjusted HRs for each risk group with covariate‐adjusted HRs and calculating the percentage change to evaluate the robustness of the group effect against clinical confounding.

### 2.7 Use of clinical disease progression models to understand change in cognition

As a secondary validation analysis, we examined whether the prognostically derived cutoffs also predicted the rate and severity of cognitive performance. Two measures of symptom severity were used: the CDR‐SoB and PACC score. Linear mixed models (LMM) were used to assess cognitive decline over time across the four groups. Baseline age, sex, years of education, and *APOE* ε4 carrier status were included as covariates to account for their effect on cognitive trajectories. Random effects (intercept and slope) were specified for individuals to model the inter‐participant variability in baseline cognitive performance and rates of cognitive change over time.

Standardized effect sizes (Cohen's d) were assessed by comparing the slope in the highest‐risk group (Aβ‐PET > 71 CL; MMSE scores < 27) with the low‐risk group (25 < Aβ‐PET ≤ 71 CL; MMSE scores ≥ 27). These statistical analyses were conducted using the *emtrends* (estimated marginal trends) functions from the *emmeans* package.^31^ As a result, the effect sizes reflect comparisons between estimated means and the differences in estimated slopes while controlling for other variables in the model. Additionally, a false discovery rate (FDR) adjustment was applied to address multiple comparisons.

## 3 RESULTS

### 3.1 Differences in clinical characteristics of participants

Prospective review of the clinical disease status over time in 454 participants from the ADNI and AIBL MCI samples (all with a baseline CDR‐Global score of 0.5 and CL > 25) identified 93 cases of clinical disease progression within 3‐years (progressors). The progressor and non‐progressor groups were well‐matched for key demographic characteristics, including age, sex, education, and study cohort. In contrast, progressors showed a significantly higher prevalence of *APOE* ε4 carriers compared with non‐progressors, indicating greater genetic risk (Table 1).

Despite the high variability in Aβ‐PET, progressors demonstrated significantly higher baseline Aβ‐PET burden compared to non‐progressors (91.7 ± 28.5 vs 73.1 ± 32.1, *p *< 0.001). At baseline, MMSE scores were slightly greater in the non‐progressors than in the progressors, despite the narrow range of cognitive values (CDR‐Global score of 0.5 and MCI diagnosis) among the included participants. Progressors had a shorter follow‐up duration and fewer follow‐up visits. This reflects the study design wherein visits following a classification of clinical disease progressions were excluded from analysis, and because withdrawal rates in each cohort became greater as clinical disease severity increased.

### 3.2 Selection of criteria based on optimal cutoffs

With the sample classified as progressors or non‐progressors, baseline levels of Aβ and MMSE scores were analyzed. Figure 1 shows Youden's Index curves across centiloid and MMSE cutoffs at 3 years for the progressors and non‐progressors. Note that different cutoffs result in different set of participants being assigned to low‐risk versus high‐risk groups. The optimal cutoff score for Aβ was 71 CL, with 56% (*n =* 254) of the participants showing Aβ‐PET values greater than this and, therefore, classified as high‐risk for clinical disease progression. For MMSE, the optimal cutoff score was 27, with 33% (*n =* 149) of the participants showing scores below this and, therefore, classified as being high‐risk.

**FIGURE 1:**
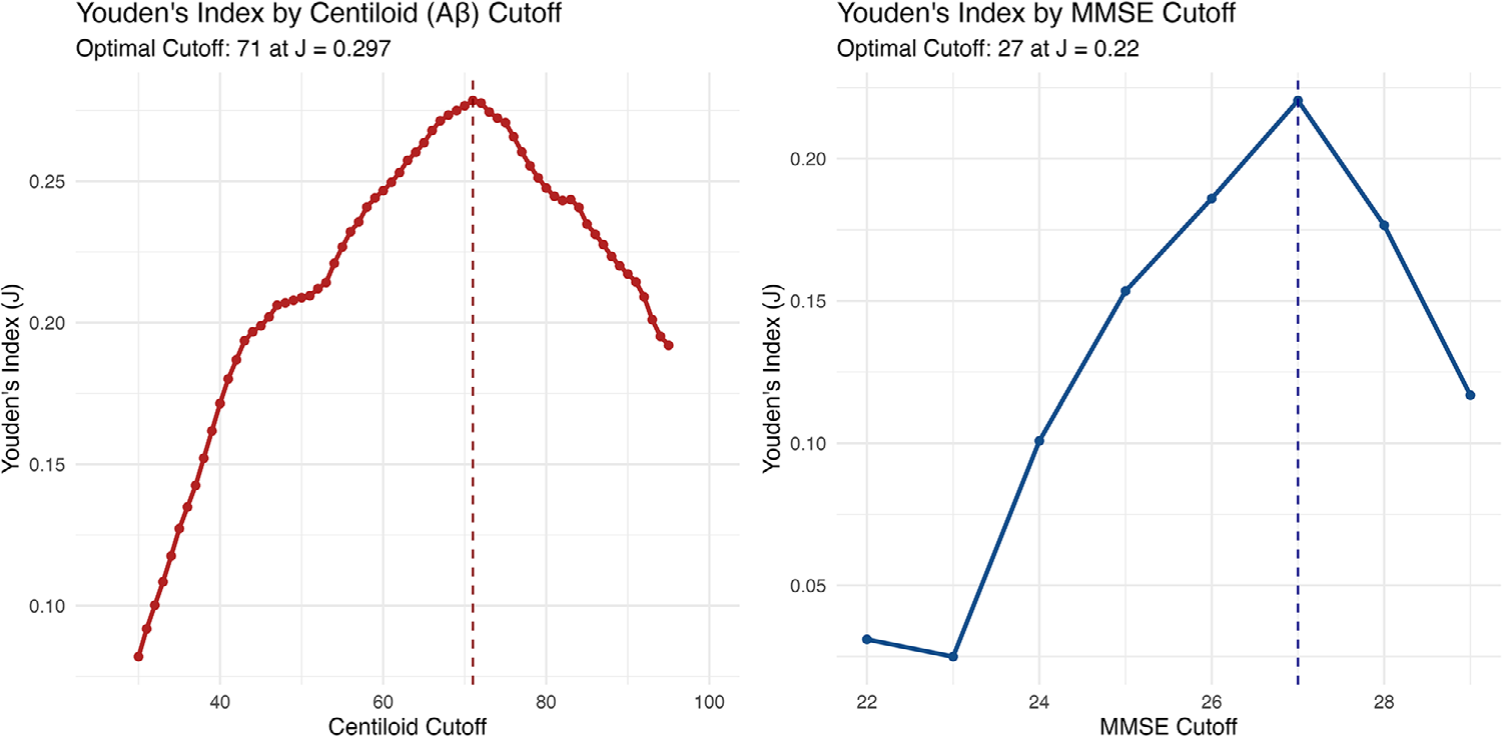
Youden's index of low‐risk vs high‐risk groups calculated for different (A) CL and (B) cognitive (MMSE) baseline values. The CL curve is smoothed over 10‐CL windows for visualisation purposes.CL, Centiloid; MMSE, Mini‐Mental State Examination.

Combination of the selected optimal cutoffs for Aβ‐PET and MMSE score yielded four risk groups, these were: (1) individuals with 25 < Aβ‐PET ≤ 71 CL and MMSE scores ≥ 27 were classified as low‐risk and treated as reference group for further analyses, (2) individuals with 25 < Aβ‐PET ≤ 71 CL and MMSE scores  < 27 were classified as intermediate‐risk with impaired‐cognition, (3) individuals with Aβ‐PET > 71 CL and MMSE scores ≥ 27 were classified as intermediate‐risk with high Aβ, and (4) the ones with Aβ‐PET > 71 CL and MMSE scores < 27 were classified as highest‐risk.

Bootstrap analysis was performed to assess the internal stability of the identified cutoffs. Results demonstrated the centiloid cutoff shows a prominent peak at the original value of 71CL (frequency ∼450), indicating this cutoff is frequently recovered across resampled datasets. A 95% CI (54.9–89) spanning 34.1 CL units, revealed substantial uncertainty. This pattern suggests that while 71 CL is the most probable optimal cutoff, considerable sampling variability exists, with non‐negligible probability mass distributed across a wide range of alternative cutoffs. The combination of a strong mode and wide CI indicates moderate internal stability but also sensitivity to sample composition. In contrast, the cutoff for the mental status score showed excellent generalizability (95% CI: 25–28), with an extremely narrow three‐unit CI and a highly concentrated unimodal distribution (peak frequency ∼650 at 27). This indicates the MMSE cutoff was highly stable across bootstrap resamples, although independent validation remains necessary.

Once these criteria were developed using the 3‐year classification of individuals into risk groups, we assessed how these groups performed over a longer follow‐up period (up to seven years).

### 3.3 Clinical disease progression risk: Aβ‐PET, MMSE, and combined Aβ‐PET/MMSE stratification

Stratification by Aβ‐PET status alone indicated that 19 of 200 participants with 25 < Aβ‐PET ≤ 71 CL (PR = 11.7%) and 74 of 254 participants with Aβ‐PET > 71 CL (PR = 36.7%) progressed to AD during the 3‐year follow‐up period. Stratification by MMSE score alone showed that 49 of 305 participants with MMSE scores ≥ 27 (PR = 20.0%) and 44 of 149 participants with MMSE scores < 27 (PR = 36.8%) progressed to AD over the same period.

Combined Aβ‐PET/MMSE stratification demonstrated a gradient of progression risk: 10 of 146 participants in the low‐risk group (25 < Aβ‐PET ≤ 71 CL; MMSE scores ≥ 27) [PR = 8.7%], nine of 54 in the intermediate‐risk with impaired‐cognition (25 < Aβ‐PET ≤ 71 CL; MMSE scores < 27) [PR = 19.8%], 39 of 159 in the intermediate‐risk with high Aβ (Aβ‐PET > 71 CL; MMSE scores ≥ 27) [PR = 30.7%], and 35 of 95 in the highest‐risk group (Aβ‐PET > 71 CL; MMSE scores < 27) [PR = 47.8%] progressed to AD during the 3‐year follow‐up (see Table S1 for more details about characteristic of the four groups). Overall, 93 of 454 participants progressed within 3 years, corresponding to an unadjusted progression proportion of 20.5%; the censoring‐adjusted 3‐year progression probability was 25.3%.

Kaplan–Meier survival curves stratified by CL and MMSE group over 7 years of follow‐up are presented in Figure 2A and 2B, respectively. Figure 2c displays survival curves for the four combined biomarker groups. Cox proportional hazards models, adjusted for age, education, sex, and *APOE* ε4 status, demonstrated that participants with MMSE scores < 27 had 2.27‐fold increased risk of progression to dementia (95% CI: 1.62–3.18, *p <* 0.001) compared to those with MMSE scores ≥ 27. Participants with Aβ‐PET > 71 CL exhibited 2.67‐fold increased risk (95% CI: 1.85–3.85, *p <* 0.001) relative to those with 25 < Aβ‐PET ≤ 71 CL.

**FIGURE 2:**
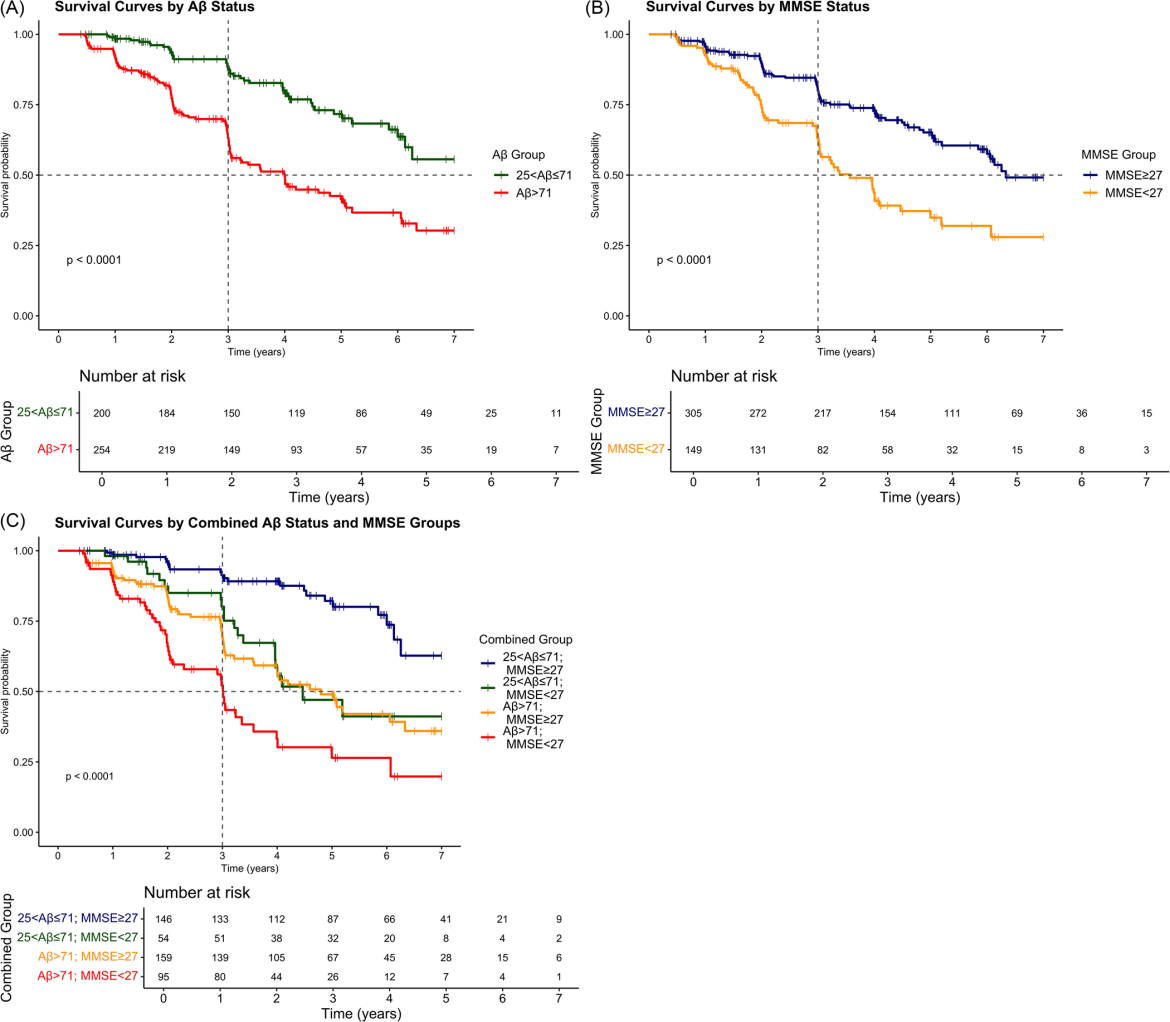
Kaplan–Meier survival curves and number of participants at risk across different stratification approaches. (A) Stratified by CL cutoff alone (71 CL): low Aβ‐PET (25 < Aβ‐PET≤71 CL, green) vs. high Aβ‐PET (Aβ‐PET > 71 CL, red). (B) Stratified by MMSE cutoff alone (score of 27): preserved‐cognition (MMSE scores ≥ 27, blue) vs. impaired‐cognition (MMSE scores < 27, orange). (C) Stratified by combined Aβ‐PET/MMSE cutoffs: low‐risk (25 < Aβ‐PET ≤ 71 CL; MMSE scores ≥ 27, blue), intermediate‐risk with impaired‐cognition (25 < Aβ‐PET ≤ 71 CL; MMSE scores < 27, green), intermediate‐risk with high Aβ‐PET (Aβ‐PET > 71 CL; MMSE scores ≥ 27, orange), and highest‐risk (Aβ‐PET > 71 CL; MMSE scores < 27, red). Aβ‐PET, amyloid‐β–positron emission tomography; CL, Centiloid; MMSE, Mini‐Mental State Examination.

Combined Aβ‐PET/MMSE analysis revealed that the highest‐risk group (Aβ‐PET > 71 CL; MMSE scores < 27) demonstrated 6.39‐fold increased risk (95% CI: 3.73–10.93, *p <* 0.001) compared to the reference group (25 < Aβ‐PET ≤ 71 CL; MMSE scores ≥ 27). The proportional hazards assumption was tested using Schoenfeld residuals and was satisfied for our grouping variable (*p =* 0.230), confirming the HR between groups is constant over time. Note that the reported HRs are adjusted for covariates (i.e., the risk difference between groups if two people had the exact same age, education, sex, *APOE* ε4 status); however, the Cox proportional hazards model can be used to estimate the survival probability for an individual or new subject too (see Supplementary Materials for details). Cox proportional hazards results also showed that Aβ‐PET/MMSE risk grouping is by far the strongest predictor (Figure S1). Among clinical covariates, only *APOE* ε4 status and education were independently statistically significant. *APOE* ε4 carriers had 71% higher risk of progression. Each additional year of education is associated with ∼7% higher hazard.

To address the known ceiling effects and limited sensitivity of MMSE in prodromal AD, we repeated cutoff‐optimization and survival analyses using California Verbal Learning Test (CVLT) delayed, a domain‐specific episodic‐memory measure. This sensitivity analysis showed a similar ordered risk gradient and comparable highest‐risk estimate (Figures S2 and S3).

In cohort‐stratified sensitivity analyses, AIBL participants had fewer and less frequent visits than ADNI participants, although observed follow‐up duration did not differ significantly. At the 3‐year, the pooled MMSE cutoff (< 27) predicted progression in both cohorts (AIBL HR = 4.38, 95% CI 1.71–11.23; ADNI HR = 3.50, 95% CI 2.18–5.63), with no evidence that this association differed by cohort (interaction *p =* 0.943). The pooled CL cutoff (> 71) was associated with progression in ADNI but not statistically significantly in AIBL; however, the cohort‐by‐CL interaction was not significant (*p =* 0.108). Full details of visit schedules, independently derived cutoffs, cohort‐specific models, and interaction tests are provided in the Supplementary Materials and Tables S2, S4.

### 3.4 Validation analysis: independent prognostic value of risk groups

The addition of the four‐group stratification significantly improved model fit over a model containing standard demographic covariates alone (LRT, *p <* 0.001). In addition, the percent change in HRs between the unadjusted models and those adjusted for demographic factors (age, sex, *APOE* ε4 status, and years of education) was small (ranging from ‐1.4% to +5.0%).

### 3.5 Validation analysis: symptomatic worsening across risk groups measured using continuous measures of disease symptoms

Analysis of longitudinal cognitive decline revealed that the rates of cognitive decline differed across the four risk groups. On the CDR‐SoB score (Figure 3a), the highest‐risk group (Aβ‐PET > 71 CL; MMSE scores < 27) exhibited the fastest rate of decline (slope = 1.15, standard error [SE] = 0.077, *p <* 0.0001), significantly exceeding all other subgroups. The intermediate‐risk with high Aβ (Aβ‐PET > 71 CL; MMSE scores ≥ 27, slope = 0.75, SE = 0.057, *p <* 0.0001) and intermediate‐risk with impaired‐cognition (25 < Aβ‐PET ≤ 71 CL; MMSE scores < 27, slope = 0.62, SE = 0.091, *p <* 0.0001) groups showed intermediate decline, whereas the low‐risk group (25 < Aβ‐PET ≤ 71 CL; MMSE scores ≥ 27, slope = 0.22, SE = 0.057, *p =* 0.0001) declined most slowly. Pairwise contrasts confirmed that the highest‐risk group differed significantly from all other subgroups (all *p <* 0.005), with a large effect size compared to the low‐risk group (*d =* 1.31).

**FIGURE 3:**
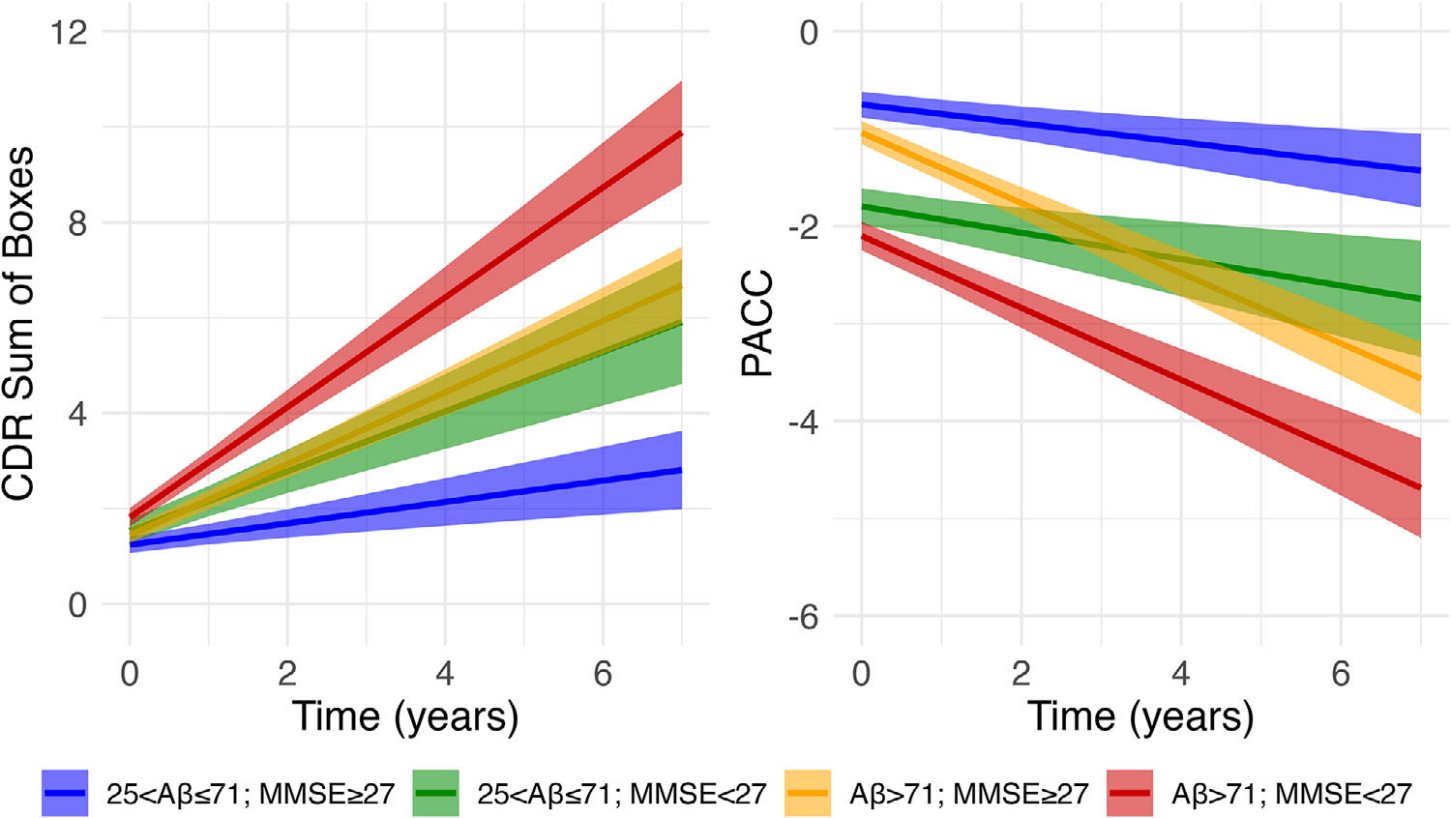
Comparison of cognitive decline across risk groups. Cognitive trajectories of (A) CDR‐SoB and (B) PACC, over 7 years across the four risk groups: low‐risk (25 < Aβ‐PET ≤ 71 CL; MMSE scores ≥ 27, blue), intermediate‐risk with impaired‐cognition (25 < Aβ‐PET ≤ 71 CL; MMSE scores < 27, green), intermediate‐risk with high Aβ‐PET (Aβ‐PET > 71 CL; MMSE scores ≥ 27, orange), and highest‐risk (Aβ‐PET > 71 CL; MMSE scores < 27, red). Aβ‐PET, amyloid‐β–positron emission tomography; CDR‐SoB, Clinical Dementia Rating Sum of Boxes; CL, Centiloid; MMSE, Mini‐Mental State Examination

For the PACC score (Figure 3b), the steepest decline was observed in the highest‐risk group (Aβ‐PET > 71CL; MMSE scores < 27, slope = ‐0.37, SE = 0.036, *p <* 0.0001), followed by the intermediate‐risk with high Aβ (Aβ‐PET > 71 CL; MMSE scores ≥ 27, slope = ‐0.36, SE = 0.025, *p <* 0.0001) and intermediate‐risk with impaired‐cognition (25 < Aβ‐PET ≤ 71 CL; MMSEscores < 27, slope = ‐0.14, SE = 0.040, *p =* 0.0008) groups. Slowest decline occurred in the low‐risk group (25 < Aβ‐PET ≤ 71 CL; MMSE scores ≥ 27, slope = ‐0.10, SE = 0.024, *p =* 0.0001). Pairwise contrasts largely reflected the same pattern, with the difference in PACC slope between the intermediate‐risk with high‐Aβ and highest‐risk group (estimate = 0.01, SE = 0.043, *p =* 0.996) not statistically significant. The highest‐risk group nevertheless showed a moderate effect size compared to the low‐risk group (*d =* 0.63), suggesting accelerated decline in participants with both high Aβ burden and impaired cognition. In sensitivity analyses using executive function and episodic memory cognitive composites (MMSE‐free cognitive composite scores), the overall pattern of group differences was similar to the ones observed in PACC (Figure S4 and Figure 3).

## 4 DISCUSSION

In this large cohort of Aβ‐PET positive MCI participants from the AIBL and ADNI studies, optimal cutoffs were identified for Aβ burden determined from PET (71 CL) and mental status, determined from the MMSE (score of 27), each providing accurate information about risk of clinical disease progression from MCI to mild AD. Combining biomarker and mental status cutoffs improved risk estimation compared with either approach alone. This interaction translated into clinically meaningful differences in 3‐year progression rates.

The intermediate‐risk groups clarified contributions of Aβ burden and mental status. Participants in the intermediate‐risk group with high Aβ‐PET (Aβ‐PET > 71 CL; MMSE score ≥ 27) showed a 30.7% 3‐year progression rate, whereas those with lower Aβ‐PET but impaired cognition (25 < Aβ‐PET ≤ 71 CL; MMSE scores < 27) demonstrated a 19.8% rate, indicating high‐risk Aβ‐PET better predicted progression than high‐risk mental status. This heterogeneity underscores why cognition alone, despite comparable HRs to Aβ‐PET burden, is insufficient for biomarker‐free risk stratification. The low‐risk group defined by MMSE score of ≥27 alone includes 52% of participants with high‐risk Aβ‐PET burden, many at elevated risk (30.7% progression), undermining its validity as a clinical trial reference group or predictor of high risk.

Risk stratification criteria were not confounded by demographic characteristics such as age, sex, *APOE* ε4 status or education. LRT testing demonstrated that an expanded model incorporating the Aβ‐PET/MMSE risk groups fitted significantly better than one containing only clinical variables, indicating prognostic information beyond clinical factors. Although all participants were Aβ‐PET positive MCI with CDR‐Global score = 0.5, this clinical category likely includes individuals at different positions along the AD timeline. Therefore, the cutoffs should be interpreted as prognostic disease‐stage markers within prodromal AD. Higher Aβ‐PET burden and lower MMSE may indicate more advanced disease stage and proximity to clinical progression, rather than a distinct progression‐specific biological mechanism. Given the MMSE's ceiling effects and limited sensitivity in prodromal populations, we repeated the analysis using CVLT delayed (Figure S2). The similar ordered risk gradient and comparable highest risk estimate support the robustness of biomarker/cognitive stratification beyond MMSE score (Figure S3).

The consistency of the criteria for predicting clinical disease progression (CDR‐Global status) and symptom worsening supports their validity. For continues measures of AD symptoms, the CDR‐SoB scale showed a stepwise hierarchy of decline, with the highest‐risk group showing the steepest negative trajectory, exceeding graded declines in intermediate and low‐risk groups. In contrast, decline on the cognitive composite was predicted more accurately by level of Aβ‐PET burden than mental status examination. All groups with elevated Aβ‐PET burden displayed rapid and identical rates of cognitive decline regardless of their initial level of cognitive impairment, suggesting that, once Aβ‐PET burden exceeds a critical threshold, it becomes the primary predictor of decline on composite cognitive measures. These consistent findings across different outcome measures strengthen confidence that the identified cutoffs capture meaningful differences in disease risk. AD symptoms extend beyond CDR‐SoB and PACC,^32^ and sensitivity analyses showed that these cutoffs predict changes in other cognitive domains, for example, executive function and episodic memory.

Bootstrap and cohort sub‐sample analyses demonstrated exceptional stability for MMSE but a moderate generalizability for Aβ‐PET CL. This likely reflects the homogeneous distribution of cognitive scores within the MCI range (with CDR‐Global score of 0.5) compared to the more heterogeneous, right‐skewed distribution of Aβ‐PET burden. The 71 CL threshold should be interpreted as a dataset‐derived prognostic split for 3‐year clinical progression within Aβ‐positive MCI, rather than as a definitive biological disease‐transition threshold. Its broad bootstrap CI indicates uncertainty around the exact value, and external validation is required before this cutoff, the MMSE score of 27 threshold, and the combined Aβ‐PET/MMSE risk groups can be considered clinically robust.

The results of this study have important clinical implications for predicting AD progression. The risk profiles developed could assist clinicians in tailoring care plans for individuals with MCI. Patients in the highest‐risk subgroup, those with *APOE* ε4 carriage, or higher education, may benefit from more frequent cognitive monitoring, earlier referral to specialist services, and carer support and advance care planning. Conversely, individuals at lower predicted risk may be managed with less intensive surveillance, enabling more efficient allocation of healthcare resources. As anti‐Aβ therapies become available, biomarker‐cognitive stratification may help identify patients most likely to benefit, optimizing resource allocation and minimizing exposure to interventions with significant costs, monitoring requirements, and potential adverse effects. From an ethical standpoint, accurate risk stratification enables informed consent, respects participant autonomy, promotes beneficence by focusing interventions on those most likely to benefit, and advances justice by ensuring appropriate distribution of research burdens and benefits. As Bunnik et al.^33^ emphasized, transparent risk disclosure in clinical research is an ethical imperative that must be balanced with appropriate support and counselling, particularly as disclosure of high‐risk status may cause psychological distress.

These results also have implications for clinical trial design. The lower censoring‐adjusted 3‐year progression rate in the current cohort (PR = 25.3%) compared with 40–50% reported in some MCI cohorts likely reflects the inclusion of early MCI cases (CDR‐Global score of 0.5 with MMSE score ≥ 20) and differences in the study design. For instance, Hyemin Jang et al.^34^ reported a 40% (74 of 112) risk of progression to AD dementia within 3 years for ADNI MCI patients; however, their analyses excluded participants followed for less than 3 years without progression of disease. Applying this exclusion criteria to this ADNI cohort yielded a higher progression rate (PR = 32%; and 77 out of 241), suggesting that differences in analytical approaches, the handling of censored observations and variable follow‐up duration, substantially influence reported progression rates. The use of Cox proportional hazards models accounts for censoring and time‐varying risk, providing more robust estimates than simple unadjusted proportions. These differences emphasize the need for participant selection for each trial, as heterogeneity increases sample‐size requirements and the likelihood of null findings, even for effective interventions. The biomarker–cognitive stratification developed here identifies a very‐high‐risk subgroup with a 47.8% 3‐year progression rate, almost doubling the cohort rate, which may improve trial efficiency by enriching for participants at higher near‐term risk. Beyond efficiency gains, targeting individuals at higher risk^35^, ^36^ may reduce participant exposure to ineffective interventions and associated burdens, shorten trial durations, and improve interpretability by reducing heterogeneity in disease trajectories. However, enrichment strategies should be tailored to trial objectives. These findings also align with existing literature demonstrating the prognostic value of combined biomarker‐cognitive assessment, including the importance of quantifying brain Aβ‐PET levels for participant selection,^37^ cognitive assessments for progression risk,^38^, ^39^ and early cognitive decline for understanding AD progression.^38^

This study has several strengths, including a large sample (454 participants) from two well‐characterized, independent cohorts spanning two continents; harmonized cognitive measures; longitudinal follow‐up for up to 7 years; cutoff optimization with bootstrap validation (1000 iterations); CL‐standardized Aβ‐PET quantification; and adjustment for established risk factors with validation of model assumptions. However, several limitations warrant consideration. The predominantly research‐volunteer composition with relatively high education levels and limited racial/ethnic diversity may limit generalizability to community‐based populations. The CL cutoff of 71, while optimal in the pooled dataset, showed substantial bootstrap variability and requires validation in independent cohorts. Model‐performance metrics were evaluated in the same dataset used for cutoff derivation and, therefore, should be interpreted as internal performance. Cohort‐stratified analyses showed no statistically significant cutoff‐by‐cohort interaction at 3 years; however, independently derived cutoffs varied between cohorts, particularly for Centiloid. Cohort‐specific calibration may therefore be appropriate for external populations. The analysis did not incorporate tau biomarkers, which limits biological interpretation. Because tau‐PET is strongly associated with clinical progression and may better reflect disease stage than amyloid burden alone,^40^ the highest‐risk Aβ‐PET/MMSE group may partly reflect greater underlying tau pathology. Plasma p‐tau biomarkers may provide scalable complementary information for biological staging, including PET‐defined staging,^41^ although their incremental prognostic value over PET‐based models varies across cohorts and disease stages.^42^ Finally, while cognitive harmonization was rigorous, it may introduce measurement error affecting cutoff precision.

In conclusion, combined Aβ‐PET and MMSE assessment provides superior prognostic stratification in MCI compared with either modality alone, identifying a very‐high‐risk subgroup with 47.8% 3‐year progression risk and 6.39‐fold increased hazard. The positive interaction between high Aβ‐PET burden and cognitive impairment suggests that individuals with both elevated amyloid burden and lower baseline cognition may have a more advanced disease stage and closer proximity to clinical progression. Longitudinal trajectories revealed that Aβ‐PET burden primarily drives decline on sensitive cognitive composites, while functional decline is more strongly modulated by the interaction of Aβ‐PET burden and baseline cognitive capacity. From a clinical trial perspective, biomarker‐cognitive enrichment may improve efficiency by identifying individuals at higher near‐term risk of progression, although formal trial‐enrichment simulations are required to quantify potential sample‐size reductions. Clinically, accurate risk stratification enables informed decision‐making about prognosis and treatment options. As the field moves toward precision medicine in AD, future work should extend clinically interpretable risk strata toward AI‐driven personalized disease‐progression models integrating continuous biomarker, cognitive, genetic, and clinical data to support individualized prognosis, clinical care, and therapeutic development. Future research should validate these cutoffs in independent, more diverse cohorts, and integrate emerging plasma biomarkers (particularly p‐tau217) with imaging and cognitive measures to enable accessible screening.

## CONFLICT OF INTEREST STATEMENT

C.L.M. reports Ad Hoc consultancy speaking engagements and scientific advice with Actinogen, Acumen, Alterity, Biogen, Eisai, Eli‐Lilly, Roche. CCR has received research grants from National Health and Medical Research Council (NHMRC) of Australia, Medical Research Future Fund (MRFF), Enigma Australia, Biogen, Eisai, Roche, and Abbvie. He is on the scientific advisory board for Enigma Biomedical Group and consulted for Prothena, Roche, Eisai, Eisai Australia, Eli Lilly Australia, Novo Nordisk Australia. M.W. serves on Editorial Boards for Alzheimer's & Dementia, and the Journal for Prevention of Alzheimer's Disease (JPAD). He has served on Advisory Boards for Acumen Pharmaceutical, Alzheon, Inc., Amsterdam UMC; MIRIADE, Cerecin, Merck Sharp & Dohme Corp., NC Registry for Brain Health, ProMIS Neurosciences, Inc., and REGEnLIFE. He also serves on the USC ACTC grant which receives funding from Eisai. M.W. has provided consulting to Acadia Pharmaceuticals, Boxer Capital, LLC, Cerecin, Inc., Clario, Dementia Society of Japan, Dolby Family Ventures, Eisai, Guidepoint, Health and Wellness Partners, Indiana University, LCN Consulting, MEDA Corp., Merck Sharp & Dohme Corp., NC Registry for Brain Health, NovoNordisk, Owkin France, ProMIS Neurosciences, Prova Education, Sai MedPartners, T3D Therapeutics, University of Southern California (USC), and WebMD. M.W. has acted as a speaker/lecturer for BrightFocus Foundation, China Association for Alzheimer's Disease (CAAD) and Taipei Medical University, as well as a speaker/lecturer with academic travel funding provided by: AD/PD Congress, Amsterdam UMC, Banner Health, Cleveland Clinic, CTAD Congress, Foundation of Learning; Health Society (Japan), Kenes, U. Madison Wisconsin, U. Penn, U. Toulouse, Japan Society for Dementia Research, Korean Dementia Society, Merck Sharp & Dohme Corp., National Center for Geriatrics and Gerontology (NCGG; Japan), University of Madison Wisconsin, University of Southern California (USC). M.W. holds stock options with Alzeca, Alzheon, Inc., ALZpath, Inc., and Anven. The other authors have nothing to disclose. Author disclosures are available in the Supporting Information.

## CONSENT STATEMENT

All AIBL participants gave written consent for publication of de‐identified data. All ADNI participants signed written informed consent for participation in the ADNI, as approved by the institutional revies board at each participating center.

## Supporting information



Supporting Information

Supporting Information

## Data Availability

A software application implementing the proposed harmonization methodology is available upon reasonable request from the corresponding author (rosita.shishegar@csiro.au).
