# Supplementary material for: Optimized amyloid‐β PET centiloid and cognitive cutoffs predict progression from mild cognitive impairment to Alzheimer's dementia
Source: Alzheimers Dement. 2026 Nov 5;22(11):e71892. doi: 10.1002/alz.71892 (PMC13740349; doi:10.1002/alz.71892)
Supplement: Supplementary file 2 [file ALZ-22-e71892-s001.docx]

**Optimized Amyloid-β PET Centiloid and Cognitive Cutoffs Predict Progression from Mild Cognitive Impairment to Alzheimer’s Dementia**

Rosita Shishegar^1,2*^, Vincent Doré^1^, Pierrick Bourgeat^3^, James D. Doecke^3,4^, Rodrigo Cánovas^1^, Simon M. Laws^4,5^, Tenielle Porter^4,5^, Michael Weiner^6^ , Colin L. Masters^7^, Hamid R. Sohrabi^8,9^, Jurgen Fripp^3^, Paul Maruff^10^, Victor L. Villemagne^11^, Christopher C. Rowe^12,13^ for the Alzheimer’s Disease Neuroimaging Initiative and the Australian Imaging Biomarkers and Lifestyle flagship study of ageing

1. The Australian e-Health Research Centre, CSIRO, Melbourne, VIC 3052, Australia;
2. School of Computing Technologies, RMIT University, Melbourne, VIC 3000, Australia;
3. The Australian e-Health Research Centre, CSIRO, Brisbane, QLD 4029, Australia;
4. Centre for Precision Health, Edith Cowan University, Joondalup, WA 6027, Australia;
5. Collaborative Genomics and Translation Group, School of Medical and Health Sciences, Edith Cowan University, Joondalup, WA 6027, Australia;
6. Center for Imaging of Neurodegenerative Diseases, University of California-San Francisco, San Francisco, CA 94158, USA;
7. Alzheimer’s Research Australia, University of Western Australia, Nedlands, WA 6009, Australia;
8. Centre for Healthy Ageing, Health Futures Institute, Murdoch University, Murdoch, WA 6150, Australia;
9. School of Psychology, Murdoch University, Murdoch, WA 6150, Australia;
10. Cogstate Ltd, Melbourne, VIC 3004, Australia;
11. Department of Psychiatry, University of Pittsburgh, 3811 O’Hara Street, Pittsburgh, PA 15213, USA;
12. Florey Institute of Neuroscience and Mental Health, The University of Melbourne, 30 Royal Parade, Parkville, VIC 3052, Australia;
13. Department of Molecular Imaging & Therapy, Austin Health, 145 Studley Road, Heidelberg, VIC 3084, Australia;

**Supplementary Material**

Table S1. Participant Characteristics for each risk group

|  | 25<Aβ≤71 CL; MMSE≥ 27 | 25<Aβ≤71 CL; MMSE<27 | Aβ>71 CL; MMSE≥ 27 | Aβ>71 CL; MMSE<27 | P-value |
| --- | --- | --- | --- | --- | --- |
| n | 146 | 54 | 159 | 95 |  |
| Cohort, n ADNI (%) | 123 (84.2%) | 36 (66.7%) | 127 (79.9%) | 69 (72.6%) | 0.025 |
| Age, years | 73.7 ± 7.5 | 76.6 ± 7.7 | 74.8 ± 6.4 | 75.5 ± 7.0 | 0.045 |
| Sex, n (%) female | 71 (48.6%) | 17 (31.5%) | 67 (42.1%) | 42 (44.2%) | 0.181 |
| *APOE* $\varepsilon$4 – positivity, % | 78 (53.4%) | 29 (53.7%) | 105 (66.0%) | 69 (72.6%) | 0.008 |
| Education, years | 15.6 ± 3.0 | 14.3 ± 3.2 | 15.3 ± 3.2 | 14.5 ± 3.4 | 0.009 |
| Follow-up duration, years | 3.7 ± 2.1 | 3.3 ± 1.6 | 3.0 ± 2.0 | 2.3 ± 1.5 | <0.001 |
| Follow-up visits, number | 4.8 ± 2.0 | 4.1 ± 1.6 | 4.2 ± 1.8 | 3.5 ± 1.6 | <0.001 |
| Aβ, baseline value | 45.5 ± 14.4 | 51.3 ± 13.0 | 97.5 ± 18.2 | 105.3 ± 24.2 | <0.001 |
| MMSE, baseline score | 28.6 ± 1.1 | 24.9 ± 1.0 | 28.4 ± 1.0 | 24.9 ± 1.3 | <0.001 |
| Progression to dementia, % | 8.65% | 19.84% | 30.65% | 47.82% | <0.001 |

Note: Continuous variables are indicated as follows: mean ± SD; ANOVAs were used for continuous variables and chi-squared tests were used for categorical variables. Reported estimates of progression risk are derived from unadjusted population data. Individual risk estimates can be derived from the Cox model for specific patient profiles (See figure S1 as an example).

Figure S1. Effect of clinical covariates on predicted survival in the highest-risk group (high Aβ, low MMSE). Predicted survival curves from the adjusted Cox proportional hazards model for the highest-risk group, varying one covariate at a time while holding others at reference values (Female sex, *APOE* ε4 non-carrier, mean age, mean education). (A) Effect of *APOE* ε4 carrier status. (B) Effect of years of education (≤12, and >12 years). Dashed vertical line indicates the 3-year time point; dashed horizontal line indicates 50% survival probability. Mean Age≅ 75 years. Mean years of education ≅15 years.

**Individualized Risk Prediction Using the Cox Model**

The proposed Cox proportional hazards model can also be used to calculate the survival probability for a new subject. For this purpose, we first determine their group based on baseline MMSE and Aβ levels. Next, we compute the linear predictor *LP* by multiplying each covariate *X*_i_ by its corresponding estimated coefficient *β*_i_ from the model and summing these products, expressed as *LP*=*β_1_X_1_*+*β_2_X_2_*+…+ *β_p_X_p_*. This linear predictor is then used to adjust the baseline hazard function *h*_0_(t), which is estimated from the data, to obtain the individual's specific hazard function f*h*(t∣X)=*h*_0_(t)⋅e^LP^. Finally, we calculate the survival function *S*(t∣X) by integrating the adjusted hazard function over time, given by *S*(t∣X)=$e^{-\int_{0}^{t} h\left( u | X \right)du}$, yielding the probability that the new subject will survive beyond a specified time without converting to mild AD. This process allows for personalized survival predictions based on the individual's characteristics and the effects of covariates.

**Education-Related Analyses: Main Effects and Interaction Tests**

We evaluated whether years of education (YoE) independently predicted progression risk and whether education modified associations of amyloid burden or baseline cognition with progression. Cox proportional hazards models included YoE as a covariate with adjustment for age, sex, and *APOE* ε4 status. Two interaction models were tested: YoE × Amyloid (continuous centiloid-derived amyloid term) and YoE × MMSE (baseline cognitive term), with progression to CDR global 1 as outcome. Interaction significance was assessed using Wald p-values.

Both interaction terms were non-significant (YoE × Amyloid: P = 0.809; YoE × MMSE: P = 0.819), indicating that the prognostic effect of amyloid burden and baseline cognition does not differ systematically between higher and lower educated individuals. This null interaction result argues against the cognitive reserve hypothesis as currently proposed by the reviewer. Under cognitive reserve theory, one would expect highly educated individuals to tolerate greater pathology before cognitive or clinical decline becomes apparent implying that education would buffer or attenuate the hazard ratio associated with high amyloid or low MMSE. The absence of a significant interaction suggests instead that education does not operate as a protective modifier of biomarker-cognition effects in this cohort.

Although higher education was positively associated with progression hazard in adjusted models, we found no significant interaction between years of education and either Aβ-PET or MMSE-defined risk group. Thus, the education effect appeared to operate as a main-effect association rather than evidence that education modified the prognostic impact of amyloid burden or baseline cognition. We therefore interpret this finding cautiously, as it may reflect cohort structure, referral or detection patterns, cognitive reserve-related threshold effects, or residual confounding rather than a confirmed biological modifier of progression.

**Sensitivity Analyses: Cognitive Cutoff and Four-Group Risk Stratification Using CVLT Delayed Recall**

As a sensitivity analysis addressing the recognised ceiling effects and limited sensitivity of MMSE in prodromal AD, we repeated the cutoff-optimization and survival analyses using CVLT delayed recall as a domain-specific measure of episodic memory. An optimized CVLT delayed recall cutoff for three-year progression to CDR-global 1.0 was derived using the Youden index. Participants were then stratified into four groups according to Aβ-PET burden and CVLT-defined memory impairment: Aβ≤71/CVLT≥6, Aβ≤71/CVLT<6, Aβ>71/CVLT≥6, and Aβ>71/CVLT<6. Cox proportional hazards models were used to estimate progression risk across these groups, and results were compared with the primary Aβ-PET/MMSE risk stratification model.

Results: The optimized CVLT delayed recall cutoff was 6. When CVLT replaced MMSE in the combined biomarker-cognitive stratification model, a similar ordered risk gradient was observed. Compared with the reference group with lower Aβ burden and preserved CVLT performance (Aβ≤71/CVLT≥6), both the Aβ≤71/CVLT<6 group (HR = 2.80, 95% CI 1.52 -5.16, p < 0.001), and the Aβ>71/CVLT≥6 group (HR = 2.54, 95% CI 1.48-4.35, p < 0.001 ) showed significantly increased risks of progression. The highest risk was observed in participants with both elevated Aβ burden and impaired CVLT performance (Aβ>71/CVLT<6; HR = 7.12 , 95% CI 4.24 -11.96, p < 0.001). This highest-risk estimate was comparable to that observed in the primary Aβ-PET/MMSE model (HR = 6.39, 95% CI 3.73–10.93, p < 0.001), supporting the robustness of biomarker-cognitive risk stratification when using a domain-specific episodic memory measure.

Figure S2. Youden’s index of low-risk vs high-risk groups calculated for different a) centiloid; b) cognitive (CVLT) baseline values. The centiloid curve is smoothed over 10-CL windows for visualisation purposes.

Figure S3. Kaplan-Meier survival curves and number of participants at risk across different stratification approaches: a) stratified by centiloid cutoff alone (71 CL): low Aβ-PET (25<Aβ-PET≤71 CL, green) vs. high Aβ-PET (Aβ-PET>71 CL, red); b) stratified by CVLT cutoff alone (6): preserved-cognition (CVLT≥6, blue) vs. impaired-cognition (CVLT<6, orange); c) stratified by combined Aβ-PET/ CVLT cutoffs: low-risk (25<Aβ-PET≤71 CL; CVLT≥6, blue), intermediate-risk with impaired-cognition (25<Aβ-PET≤71 CL; CVLT<6, green), intermediate-risk with high Aβ-PET (Aβ-PET>71 CL; CVLT≥6, orange), and highest-risk (Aβ-PET>71 CL; CVLT<6, red).

**Sensitivity Analyses: Symptomatic Worsening Across Risk Groups Measured Using Continuous MMSE-Free Measures of Disease Symptoms**

Sensitivity analyses based on executive function and episodic memory cognitive composites (MMSE-free cognitive composite scores) demonstrated an overall pattern of group differences similar to that observed for the PACC.

Figure S4. Comparison of cognitive decline across risk groups; a) cognitive trajectories of a) executive function and b) episodic memory, over 7 years across the four risk groups: low-risk (25<Aβ-PET≤71 CL; MMSE≥27, blue), intermediate-risk with impaired-cognition (25<Aβ-PET≤71 CL; MMSE<27, green), intermediate-risk with high Aβ-PET (Aβ-PET>71 CL; MMSE≥27, orange), and highest-risk (Aβ-PET>71 CL; MMSE<27, red).

**Sensitivity Analyses: Comparison of Participants with and without Observed Progression**

To evaluate whether baseline characteristics differed by observed progression status, we conducted a prespecified sensitivity analysis in three steps. First, we compared baseline characteristics of participants with no observed event within follow-up with participants who progressed, using two-sample t-tests for continuous variables (age, education, amyloid burden, MMSE) and chi-square tests for categorical variables (sex, *APOE* ε4, study cohort; Fisher’s exact test if expected counts were small). Second, for baseline variables with significant between-group differences, we reported directionality by identifying which group (censored vs non-censored) had the higher baseline mean (continuous variables) or higher prevalence of the most differentiating category (categorical variables). Third, we evaluated group-level non-event/censoring patterns across predefined risk strata by testing differences in overall non-event/censoring and non-event/censoring before 3 years, and assessed time-to-censoring differences using both a Kruskal-Wallis test (censored participants only) and a log-rank-style test treating censoring as the event. Statistical significance was defined as p < 0.05.

Results showed significant baseline differences for amyloid, MMSE, and *APOE* ε4. Amyloid was higher in participants with observed progression (87.995 vs 71.535, p < 0.001), whereas MMSE was higher in participants without observed progression (27.641 vs 26.649, p < 0.001). *APOE* ε4 distribution also differed (p = 0.00275), with a higher prevalence of *APOE* ε4 non-carriers in the censored group. Overall non-event/censoring proportions differed across risk strata, while early non-event/censoring (before 3 years) did not. For time-to-censoring comparisons, the Kruskal-Wallis test was significant, but censoring-time curves were not significantly different in the log-rank-style analysis.

In conclusion, these findings indicate that participants with and without observed progression differed in expected directions for baseline risk markers. However, this analysis should not be interpreted as a direct test of informative dropout. However, this analysis should not be interpreted as a direct test of informative dropout. The no-observed-event group combines true loss to follow-up, administrative censoring, and stable event-free participants, and because exact withdrawal dates were unavailable. Accordingly, the primary risk estimates remain interpretable, with appropriate caution regarding potential censoring-related bias.

**Sensitivity Analyses: Cohort-stratified**

To assess whether cohort imbalance or differences in assessment schedules influenced the pooled findings, we conducted sensitivity analyses separately in AIBL and ADNI. Analyses used the sample over a maximum period of 7 years. We compared the number of visits, median interval between visits, follow-up duration, and visits per year using Wilcoxon rank-sum tests. Within each cohort, CL and MMSE cutoffs were independently derived for 3-year progression using the Youden index and 1,000 bootstrap resamples. We then estimated adjusted Cox proportional-hazards models within each cohort using the pooled thresholds (Aβ-PET >71 and MMSE <27), adjusted for age, sex, years of education, and *APOE* ε4 status. Finally, pooled Cox models included cutoff-by-cohort interaction terms to formally test heterogeneity.

The complete-case sensitivity sample comprised 99 AIBL participants (23 progression events within 3 years) and 355 ADNI participants (88 progression events within 3 years). Assessment schedules differed: AIBL had fewer and less frequent visits than ADNI, whereas observed follow-up duration did not differ significantly (Table S2). Independently derived cutoffs showed some cohort-specific variability, particularly for Centiloid, with broad bootstrap intervals in AIBL (Table S3). Nevertheless, the pooled MMSE cutoff (<27) was associated with increased progression risk in both cohorts: AIBL HR=4.38 (95% CI 1.71–11.23; *p=0.002*) and ADNI HR=1.96 (95% CI 1.27–3.02), p=0.002). The MMSE-by-cohort interaction was not significant (p=0.186). The pooled Aβ-PET cutoff (>71) was associated with progression in ADNI but not statistically significantly in AIBL; however, the Centiloid-by-cohort interaction was also not significant (p=0.099; Table S4).

Table S2. Comparison of visits and follow-up by cohort (7-year window).

| **Cohort** | **N** | **Visits, median (IQR)** | **Visit interval, years,**  **median (IQR)** | **Follow-up, years,**  **median (IQR)** | **Visits/year, median (IQR)** |
| --- | --- | --- | --- | --- | --- |
| AIBL | 99 | 3.00 (2.00–3.00) | 1.52 (1.46–1.64) | 2.98 (1.60–4.42) | 1.00 (0.88–1.25) |
| ADNI | 355 | 4.00 (3.00–6.00) | 0.78 (0.54–1.00) | 2.97 (1.75–4.08) | 1.64 (1.35–2.01) |
| Wilcoxon p value | – | <0.001 | <0.001 | 0.494 | <0.001 |

Table S3. Independently derived cohort-specific cutoffs.

| **Cohort** | **Measure** | **N** | **Events** | **Optimal cutoff** | **Youden’s J** | **Bootstrap median** | **Bootstrap 95% CI** |
| --- | --- | --- | --- | --- | --- | --- | --- |
| AIBL | Aβ-PET | 99 | 23 | 68 | 0.186 | 85 | 45–97 |
| AIBL | MMSE | 99 | 23 | 27 | 0.327 | 27 | 26–29 |
| ADNI | Aβ-PET | 355 | 88 | 71 | 0.324 | 71 | 46–88 |
| ADNI | MMSE | 355 | 88 | 28 | 0.232 | 28 | 27–29 |

Note: Cutoffs were selected by maximizing Youden’s J within each cohort. Bootstrap intervals are percentile intervals from 1,000 resamples and describe sampling variability in the selected cutoff.

Table S4. Cohort-specific associations for pooled cutoffs and pooled cohort-by-cutoff interaction tests.

| **Analysis** | **Measure / cutoff** | **N** | **Events** | **HR** | **95% CI** | **p value** | **C-index** |
| --- | --- | --- | --- | --- | --- | --- | --- |
| AIBL | Aβ-PET >71 | 99 | 23 | 2.08 | 0.82–5.27 | 0.124 | 0.607 |
| ADNI | Aβ-PET >71 | 355 | 88 | 3.95 | 2.34–6.68 | <0.001 | 0.719 |
| AIBL | MMSE <27 | 99 | 23 | 4.38 | 1.71–11.23 | 0.002 | 0.683 |
| ADNI | MMSE <27 | 355 | 88 | 1.96 | 1.27–3.02 | 0.002 | 0.659 |
| Pooled interaction | Aβ-PET >71 × cohort | 454 | 111 | 2.38 | 0.85–6.69 | 0.099 | – |
| Pooled interaction | MMSE <27 × cohort | 454 | 111 | 0.51 | 0.19–1.38 | 0.186 | – |

Note: Cohort-specific Cox models were adjusted for age, sex, years of education, and *APOE* ε4 status. Interaction HRs compare the cutoff association in ADNI relative to AIBL.

**Additional Analyses: Internal Survival Model Performance**

Because model-performance metrics were evaluated in the same dataset used for cutoff derivation, these estimates should be interpreted as internal performance rather than optimism-corrected or externally validated performance. Model performance was additionally assessed using discrimination and calibration metrics, and incremental prognostic value analyses. First, discrimination was quantified using Harrell’s C-index, which was 0.711 (95% CI 0.670 to 0.753), indicating good internal risk discrimination.

Second, we report 3-year group-level calibration by comparing observed Kaplan-Meier progression risk with Cox-model predicted risk across the four Aβ/MMSE strata. Observed progression increased monotonically across groups (8.6%, 19.8%, 30.6%, 47.8%), with small calibration gaps in lower-risk groups and modest underestimation in higher-risk groups, supporting overall calibration while indicating conservative risk estimates at the highest risk levels.

Third, we directly tested whether Aβ/MMSE staging adds information beyond standard clinical variables. The likelihood ratio test comparing nested models (clinical covariates only versus covariates plus Aβ/MMSE staging) was highly significant (P < 0.001), demonstrating substantial incremental prognostic value of the staging system.

Finally, hazard-ratio stability analyses showed minimal attenuation after adjustment for age, sex, *APOE* ε4 allele status, and education (percent change: +5.0%, -3.2%, and -1.4% across non-reference staging groups), indicating that the staging effect is robust and not primarily driven by confounding clinical covariates.

Together, these results support the apparent discrimination, incremental prognostic value, and covariate robustness of the proposed staging framework, while external validation and optimism-corrected performance estimation remain necessary to determine generalizable predictive performance.
